# MiR-145 negatively regulates Warburg effect by silencing KLF4 and PTBP1 in bladder cancer cells

**DOI:** 10.18632/oncotarget.16524

**Published:** 2017-03-23

**Authors:** Koichiro Minami, Kohei Taniguchi, Nobuhiko Sugito, Yuki Kuranaga, Teruo Inamoto, Kiyoshi Takahara, Tomoaki Takai, Yuki Yoshikawa, Satoshi Kiyama, Yukihiro Akao, Haruhito Azuma

**Affiliations:** ^1^ United Graduate School of Drug Discovery and Medical Information Sciences, Gifu University, Gifu 501-1193, Japan; ^2^ Department of Urology, Osaka Medical College, Takatsuki, Osaka 569-8686, Japan; ^3^ Department of General and Gastroenterological Surgery, Osaka Medical College, Takatsuki, Osaka 569-8686, Japan

**Keywords:** miR-145, Warburg effect, KLF4, bladder cancer

## Abstract

The Warburg effect is a well-known feature in cancer-specific metabolism. We previously reported on the role of microRNA (miR)-145 as a tumor-suppressor in human bladder cancer (BC) cells. In this study, we reveal that miR-145 decreases the Warburg effect by silencing KLF4 in BC cells. The expression levels of miR-145 were significantly lower in clinical BC samples and BC cell lines compared to those in normal tissues and HUC cells. Luciferase assay results showed that miR-145 directly bound to 3′UTR of KLF4, which was shown to be overexpressed in the clinical BC samples using Western blot analysis and immunohistochemistry. Remarkable growth inhibition and apoptosis were induced by the ectopic expression of miR-145 or by the gene silencing of KLF4 (siR-KLF4). Also, Warburg effect-related genes such as PTBP1/PKMs were regulated by the transfection of BC cells with miR-145 or siR-KLF4. These results thus indicate that the miR-145/KLF4/PTBP1/PKMs axis is one of the critical pathways that maintain the Warburg effect in BC carcinogenesis. MiR-145 perturbed the Warburg effect by suppressing the KLF4/PTBP1/PKMs pathway in BC cells, resulting in significant cell growth inhibition.

## INTRODUCTION

MicroRNAs (miRNAs) are single-stranded noncoding small RNAs that repress translation or induce degradation of target messenger RNAs (mRNAs) through binding to specific complementary sites within the 3′ untranslated region (3′-UTR) of mRNAs [1]. A growing body of evidence indicates that dysregulation of miRNA expression contributes to carcinogenesis in cancers [2, 3]. MiR-145 is one of the representative anti-oncomiRs in various kinds of cancers. We have previously reported that miR-145 is downregulated and acts as a tumor suppressor in colon adenomas [4, 5] and colon cancers [6], gastric cancers [7], chronic lymphocytic leukemias, B-cell lymphomas [8], and bladder cancer cells [9, 10]. We also reported that intravesical administration of exogenous miR-145 has an anti-tumor effect on orthotropic xenograft human bladder cancer tumors [11]. On the other hand, we recently described miRs such as miR-133b and miR-124 to regulate the Warburg effect by suppressing the PTBP1/PKM isoform axis. PKM has 2 isoforms, PKM1 and PKM2, which are produced by alternative splicing of transcripts in the PKM gene. PTBP1 induced the switching of PKM isoform expression from PKM2 to PKM1. PKM2 is exclusively expressed in embryonic, proliferating, and cancer cells, promoting glycolysis even in an aerobic environment. PKM1 is expressed in normal differentiated tissues and promotes oxidative phosphorylation [12, 13]. It is also known that miR-145 targets c-Myc [14], which regulates PTBP1, leading to impairment of the Warburg effect (PTBP1/PKMs axis). In this context, we explored the so far unaddressed function of miR-145 in the Warburg effect.

Among the targeted genes (mRNAs) of miR-145, we focused on Kruppel-like factor 4 (KLF4) in relation to the Warburg effect. As mentioned above, KLF4 is a transcription factor that is well known to be one of the essential factors of iPS cells. KLF4 mRNA expression is found primarily in postmitotic, terminally differentiated epithelial cells in organs such as skin and lungs and also in those in the gastrointestinal tract [15, 16]. KLF4 is a potential oncogene in patients with various cancers. It is reported that KLF4 also regulates the Warburg effect [12], but the precise role and underlying signaling cascade of KLF4 in the Warburg effect in bladder cancer remain unclear.

In the current study, we examined whether or not miR-145 could affect the regulation of the Warburg effect through silencing KLF4 in bladder cancer cells. We demonstrate that the expression of KLF4 was increased in bladder cancer (BC) cells from patients. In addition, the ectopic expression of miR-145 partly induced apoptosis by regulating the PTBP1/PKM1/PKM2 axis through the silencing of KLF4 in BC cells. We found that not only the miR-145/c-Myc/PTBP1/PKMs axis, but also the novel miR-145/KLF4/PTBP1/PKMs cascade operated in the regulation of cancer energy metabolism.

## RESULTS

### The expression of miR-145 was significantly decreased in clinical tumor samples from BC patients and BC cell lines

**Figure 1:**
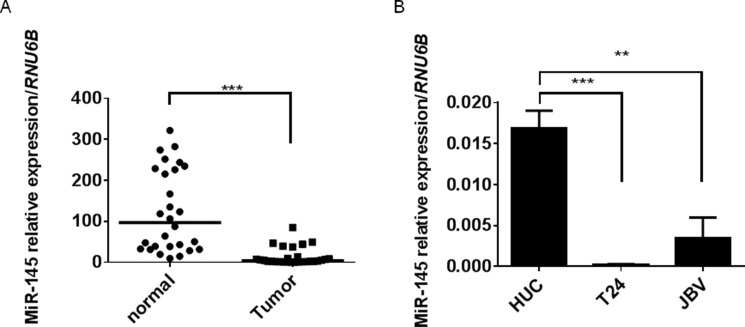
Expression of miR-145 is decreased in clinical BC-tumor samples and BC cell lines (**A**) Relative expression levels of miR-145 in clinical BCs and NBEs. (**B**) Relative expression levels of miR-145 in HUC, T24, and 253J B-V cells. Results are presented as mean ± SD; **P < 0.01; ***P < 0.001.

It was already reported by us and others that miR-145 is decreased in BC samples [17–19] and BC cell lines [20]. We thus first reconfirmed the decrease of miR-145 in BC samples by performing real-time RT-PCR. Results indicate that the expression of miR-145 were significantly decreased in BCs compared with those in the normal bladder epithelium (NBE) adjacent to the tumors (Figure 1A).

In addition, the expression levels of miR-145 were significantly decreased in the human bladder cancer T24 and 253J B-V cell lines (Figure 1B). Based on these results, we focused on the role of miR-145 in carcinogenesis of BC from the viewpoint of cancer energy metabolism.

### MiR-145 impairs the Warburg effect through the c-Myc/PTBP1/PKMs axis by silencing c-Myc

**Figure 2:**
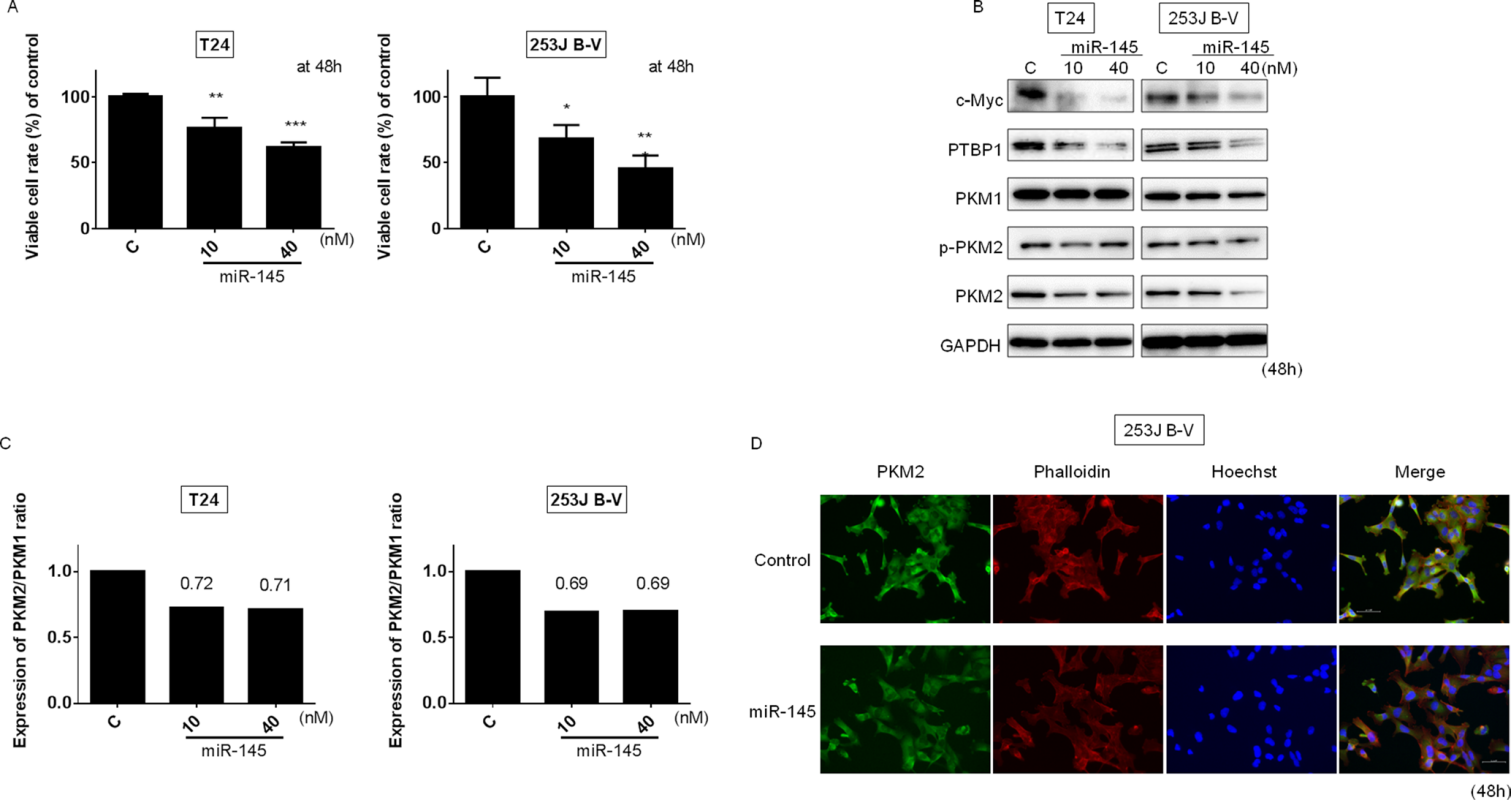
Ectopic expression of miR-145 induced growth inhibition through impaired Warburg effect Effects of ectopic expression of miR-145 on cell viability (**A**) and expression of various proteins estimated by Western blot analysis (**B**) at 48 h after transfection of T24 and 253J B-V cells with miR-145 (10, 40 nM). (**C**) PKM2/PKM1 ratio calculated based on densitometric values of PKM1 and PKM2 in “B.” Numbers represent ratios with control values taken as “1.000.” (**D**) Immunofluorescence of PKM2 (lower panels) in 253J B-V cells transfected with miR-145 (20 nM). Representative images (scale bar, 50 μm) are shown. PKM2 is stained green, cytoskeleton is stained red. Nuclei appear in blue. Results are presented as mean ± SD; *P < 0.05; **P < 0.01; ***P < 0.001.

We have previously published that ectopic expression of miR-145 led to significant growth inhibition through the suppression of PI3K/Akt and MAPK signaling pathways in bladder cancer cells [21]. As shown in Figure 2A, exogenous miR-145 also significantly suppressed the growth of T24 and 253J B-V cells. These results suggest that exogenous miR-145 functioned as an anti-oncomir in these cells. Recently, we reported that miR-124 regulates the Warburg effect through targeting PTBP1 in colon cancer cells [22, 23]. It is well known that c-Myc, which positively regulates the expression of PTBP1 in the upstream [24], is a direct target of miR-145 [14]. Therefore, we examined the effects of ectopic expression of miR-145 on the c-Myc/PTBP1 network in the BC cell lines. As a result, the introduction of miR-145 induced downregulation of c-Myc and reduced PKM2/PKM1 ratio in either cell line (Figure 2B and 2C). Furthermore, in order to further validate the switching, we performed immunofluorescence staining for PKM2 in 253J B-V cells. Figure 2D shows the decreased expression of PKM2 at the single-cell level in the cells transfected with miR-145. Taken together, these results indicate that miR-145 targets c-Myc, resulting in an impaired Warburg effect, at least in part by negatively affecting the c-Myc/PTBP1/PKMs axis.

**Figure 3:**
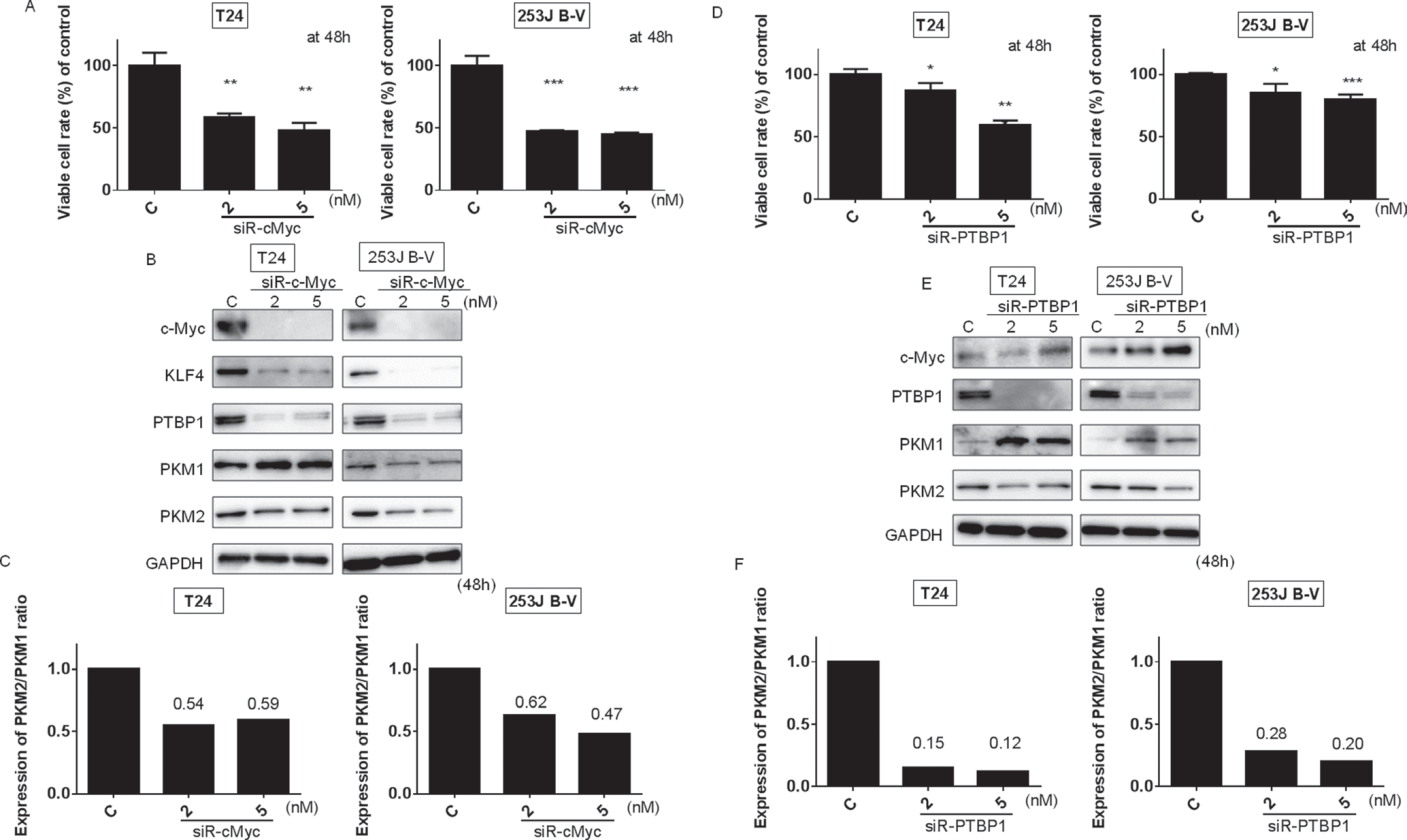
C-Myc/PTBP1 cascade regulates the Warburg effect (**A**) Effects of c-Myc knockdown on cell growth of T24 and 253J B-V cells. (**B**) Protein expression of Warburg effect-associated genes after the transfection of T24 and 253J B-V cells with siR-c-Myc. (**C**) PKM2/PKM1 ratio calculated based on densitometric values of PKM1 and PKM2 in “B.” Numbers represent ratios with control values taken as “1.000.” (**D**) Effects of PTBP1 knockdown on cell growth of T24 and 253J B-V cells. (**E**) Protein expression of Warburg effect-associated genes after the transfection of T24 and 253J B-V cells with siR-PTBP1. (**F**) PKM2/PKM1 ratio calculated based on densitometric values of PKM1 and PKM2 in “E.” Numbers represent ratios with control values taken as “1.000.” Results are presented as mean ± SD; *P < 0.05; **P < 0.01; ***P < 0.001.

We previously reported that the silencing of c-Myc decreased the transcription of PTBP1, which is a splicer of the PKM gene, and modulated the Warburg effect through the switching of PKM isoform expression from PKM2 to PKM1, thus reducing the PKM2/PKM1 ratio [22, 23, 25]. We thus set out to clarify the relationship between c-Myc and PTBP1 with respect to the Warburg effect. We examined the effect of the gene silencing of c-Myc or PTBP1 by using siR-c-Myc or siR-PTBP1, respectively, on the expression of Warburg effect-related proteins by performing Western blot analysis. As shown in Figure 3A and 3D, silencing of either of these genes induced a significant growth inhibition in both T24 and 253J B-V cells. Western blot analysis showed that the knockdown of c-Myc reduced the PKM2/PKM1 ratio through the downregulation of PTBP1 (Figure 3B and 3C). Also, knockdown of PTBP1 decreased the PKM2/PKM1 ratio (Figure 3E and 3F). These results strongly suggest that c-Myc and PTBP1 positively regulated the Warburg effect through the c-Myc/PTBP1/PKMs axis. Thus, miR-145 affected the Warburg effect through the downregulation of c-Myc, leading to the decreased PKM2/PKM1 ratio in these cell lines.

### KLF4 is a target of miR-145 in the Warburg effect in BC cells

**Figure 4:**
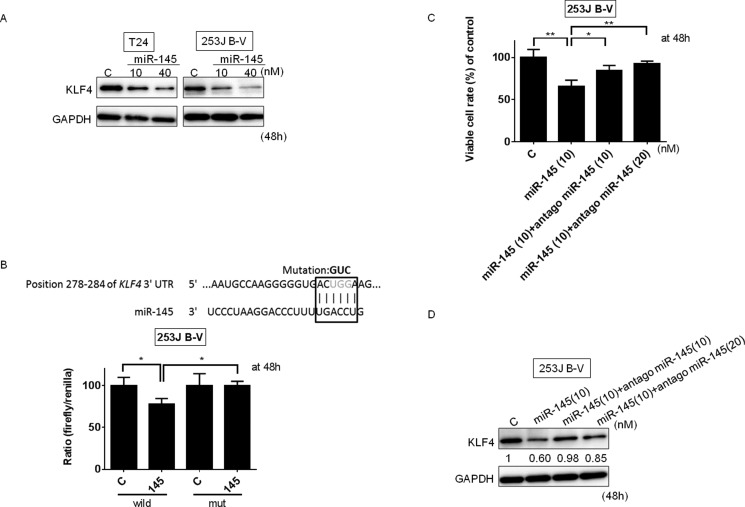
KLF4 was a target mRNA of miR-145 in BC cells (**A**) MiR-145 silenced KLF4 expression in T24 and 253J B-V cells. The protein expression of KLF4 in T24 and 253J B-V cells at 48 h after the transfection with miR-145 (10, 40 nM) is shown. (**B**) Luciferase activities after co-transfection with control or miR-145 and wild-type or mutant-type pMIR vectors having the predictive miR-145 binding site in the 3′-UTR of KLF4. The upper panel shows the region of the 3′-UTR of human KLF4 mRNA complementary to the mature miR-145. The box indicates the predicted binding sites for miR-145. (**C** and **D**) Effect of combined treatment of 253J B-V cells with antagomiR-145 and miR-145 is indicated. 253J B-V cells were transfected with non-specific control, miR-145 (10 nM), miR-145 (10 nM) + antagomiR-145 (10 nM) or miR-145 (10 nM) + antagomiR-145 (20 nM). The viable cell rate (C) and expression level of KLF4 (D) were assessed at 48 h after the transfection. Densitometric values are shown for KLF4. Results are presented as mean ± SD; *P < 0.05; **P < 0.01.

KLF4 is a zinc-finger transcription factor expressed in the epithelium of a variety of tissues including the intestinal tract, skin, cornea, and lung [15, 16, 26, 27]. Also, KLF4, being one of the Yamanaka factors, plays an important role in induced pluripotent stem (iPS) cells [28]. It was reported that KLF4 lies in the upstream of PTBP1 [12], but the mechanism is still not clearly defined. An extensive analysis in the database of Target Scan 6.2 (http://www.targetscan.org/) indicated that miR-145 targets KLF4. Therefore, we sought to clarify whether KLF4 was a direct target of miR-145 and if KLF4 affected the Warburg effect. We therefore transfected T24 and 253J B-V cells with miR-145. As shown in Figure 4A, the protein expression of KLF4 was remarkably decreased in both cells. We then performed a luciferase assay for KLF4 and confirmed that the activity of wild-type pMIR-KLF4 was significantly reduced after the introduction of miR-145 into 253J B-V cells. On the other hand, a mutation in the KLF4 3′-UTR-binding site markedly abolished the ability of miR-145 to affect the luciferase activity (Figure 4B). Furthermore, treatment with antagomiR-145 reversed the growth inhibition elicited by miR-145 and the increased expression level of KLF4 in 253J B-V cells (Figure 4C and 4D). Taken together, these results indicated that miR-145 targeted KLF4 at the translational level in BC cells.

**Figure 5:**
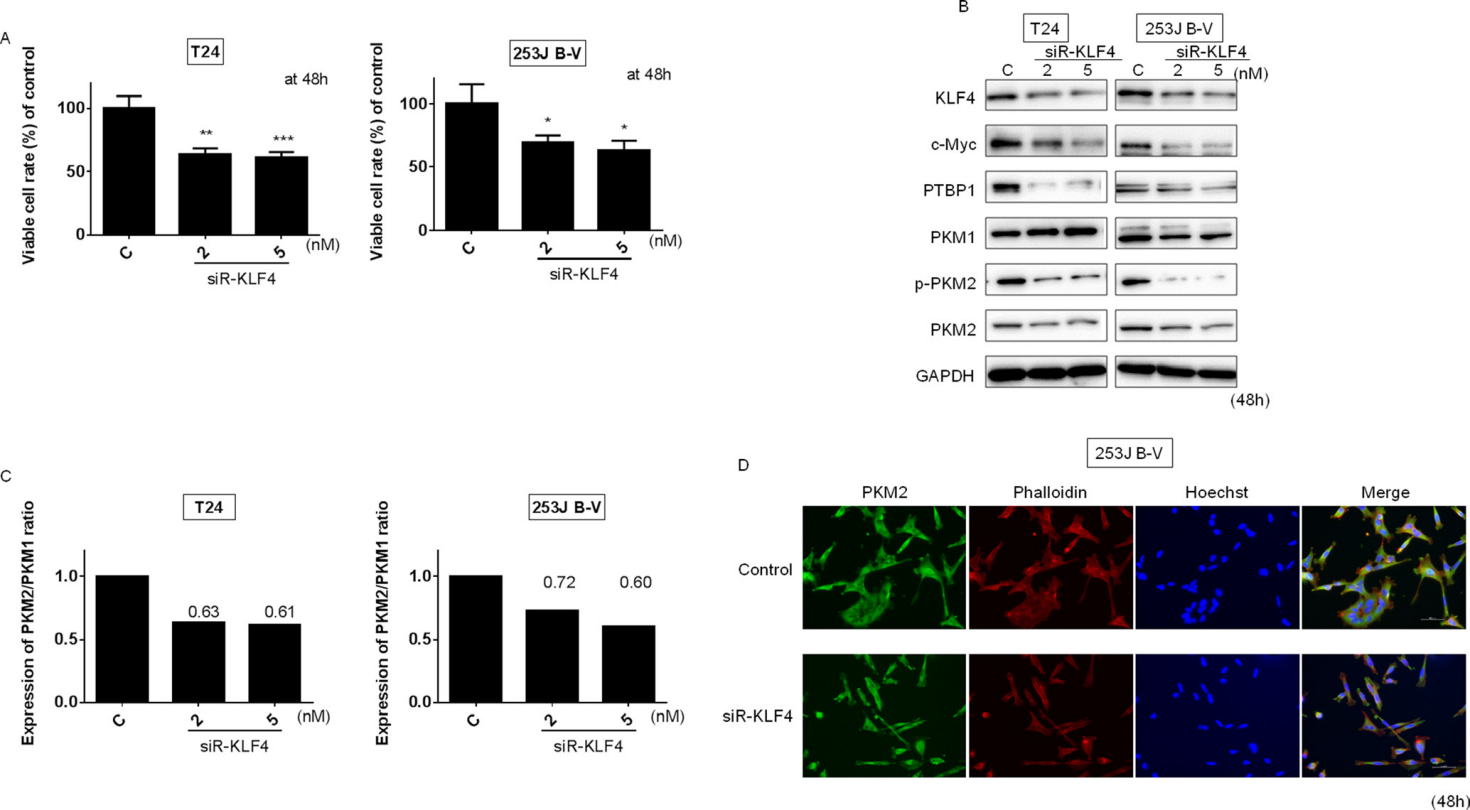
Knockdown of KLF4 affected PTBP1/PKMs cascade in BC cells Effects of gene silencing of KLF4 on cell viability (**A**) and expression of various proteins estimated by Western blot analysis (**B**) at 48 h after transfection of T24 and 253J B-V cells with siR-KLF4 (2, 5 nM). (**C**) PKM2/PKM1 ratio calculated based on densitometric values of PKM1 and PKM2 in “B.” Numbers represent ratios with control values taken as “1.000.” (**D**) Immunofluorescence of PKM2 (lower panels) in 253J B-V cells treated with siR-KLF4 (5 nM). Representative images (scale bar, 50 μm) are shown. PKM2 is stained green, the cytoskeleton is stained red. Nuclei appear in blue. Results are presented as mean ± SD; *P < 0.05; **P < 0.01; ***P < 0.001.

Next, we examined the effect of KLF4 knockdown by siRNA for KLF4 (siR-KLF4) on the PTBP1/PKM axis. As shown in Figure 5A, the silencing of KLF4 induced a significant growth inhibition in both cells. Western blot analysis showed the downregulation of PTBP1 expression (Figure 5B), resulting in a decreased PKM2/PKM1 ratio in both kinds of cells tested (Figure 5B and 5C). These results were also validated by immunocytostaining for PKM2 at the single-cell level (Figure 5D). Thus, KLF4 positively regulates Warburg effect in the upstream of PTBP1.

**Figure 6:**
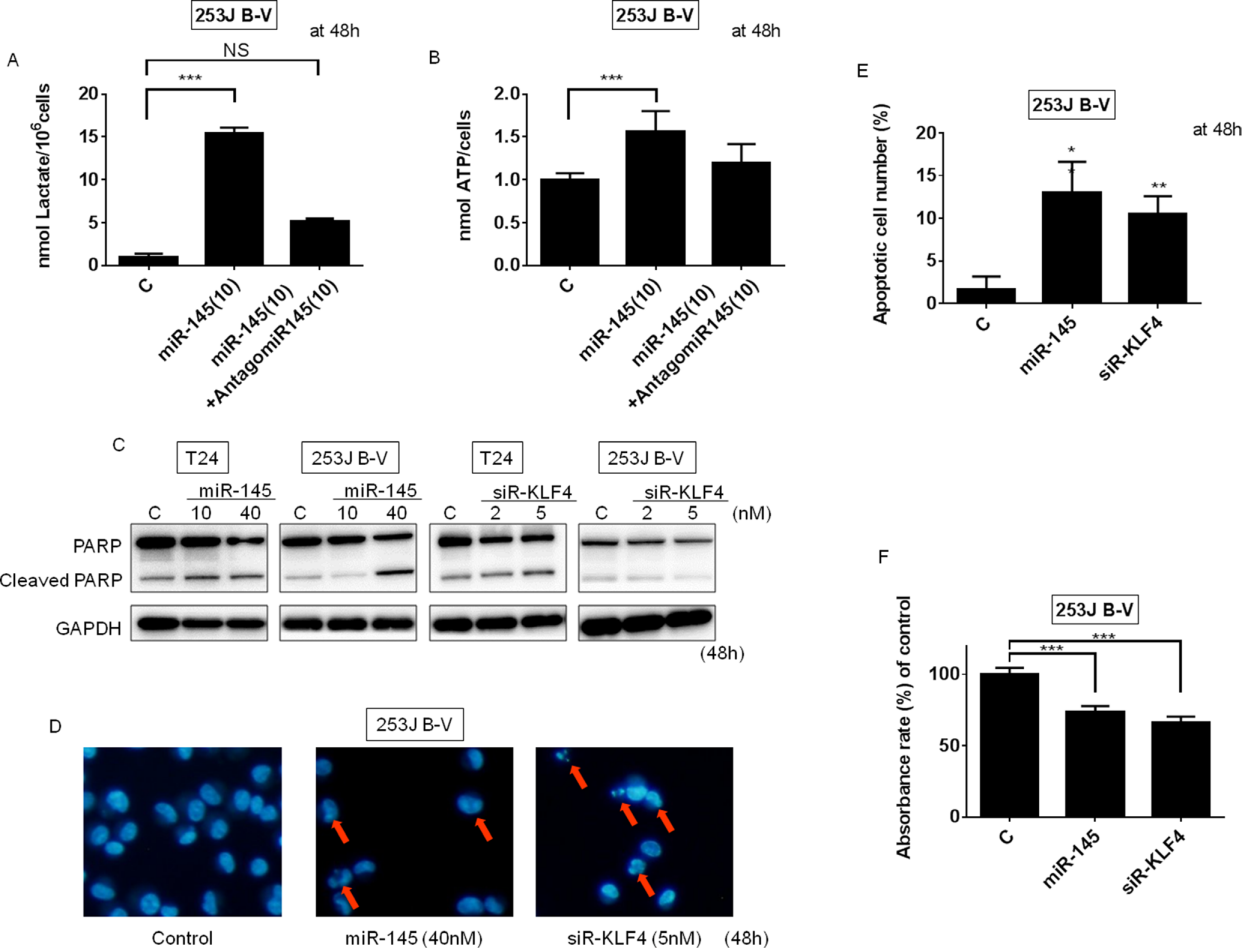
Ectopic expression of miR-145 or knockdown of KLF4 induced apoptosis in BC cells (**A**) Lactate production was measured at 48 h after the transfection of 253J B-V cells with miR-145 (10 nM) or miR-145 (10 nM) plus antagomiR-145 (10 nM). (**B**) ATP production was measured at 48 h after the transfection of 253J B-V cells with miR-145 (10 nM) or miR-145 (10 nM) plus antagomiR-145 (10 nM). (**C**) Protein expression of PARP at 48 h after the transfection of 253J B-V cells with miR-145 (10, 40 nM). (**D** and **E**) Hoechst33342 staining at 48 h after the transfection of 253J B-V cells with miR-145 (40 nM) or siR-KLF4 (5 nM). Apoptotic cells are indicated by the red arrows. (**F**) The cell viability of tumor cells within the 3D-spheroids was measured using the MTT assay. Results are presented as mean ± SD; **P < 0.01; ***P < 0.001.

We next examined the intracellular levels of lactate and ATP after switching PKM isoforms from PKM2 to PKM1 by the transfection with miR-145. The results indicated that the lactate and ATP levels were significantly increased in 253J B-V cells. These data reflected the promotion of oxidative phosphorylation by the switching of the PKMs from PKM2 to PKM1. As to the increased intracellular lactate, we hypothesize that the switching results in promotion of glycolysis even if PKM2 is decreased. Treatment with antagomiR-145 reversed the increased levels of lactate and ATP (Figure 6A and 6B).

### Ectopic expression of miR-145 or knockdown of KLF4 induces apoptosis in BC cells

We previously reported that ectopic expression of miR-145 induces apoptosis through the downregulation of c-Myc and socs7 in bladder cancer cells [9–11]. We thus confirmed this effect by performing assays to evaluate apoptotic cell death in T24 and 253J B-V cells after the transfection of these cells with miR-145 or siR-KLF4. As expected, the amount of cleaved PARP tended to increase in the miR-145-transfected cells (Figure 6C). Hoechst33342 nuclear staining showed the typical apoptotic features, such as condensed chromatin and nuclear fragmentation, in the miR-145-treated 253J B-V cells (Figure 6D and 6E). On the other hand, the knockdown of KLF4 induced apoptosis in some of the 253J B-V cells, as assessed by Western blotting of PARP and Hoechst33342 nuclear staining (Figure 6C–6E). Taken together, these findings demonstrate that transfection with miR-145 or siR-KLF4 negatively contributes to cell proliferation and induced apoptosis.

### Growth inhibition of 3-D tumor spheroids by treatment either with miR-145 or siR-KLF4

In order to examine whether miR-145 or siR-KLF4 could suppress the growth of solid tumors, we used the *in vitro* 3D spheroid system. Although the spheroids of 253J B-V cells in the control group exhibited a rapid growth, the growth of the tumor cells in the spheroids was significantly suppressed by treatment with either miR-145 or siR-KLF4 (100 nM; Figure 6F).

### Increased expression of KLF4, PTBP1, and PKM2 in clinical bladder cancer samples from patients

**Figure 7:**
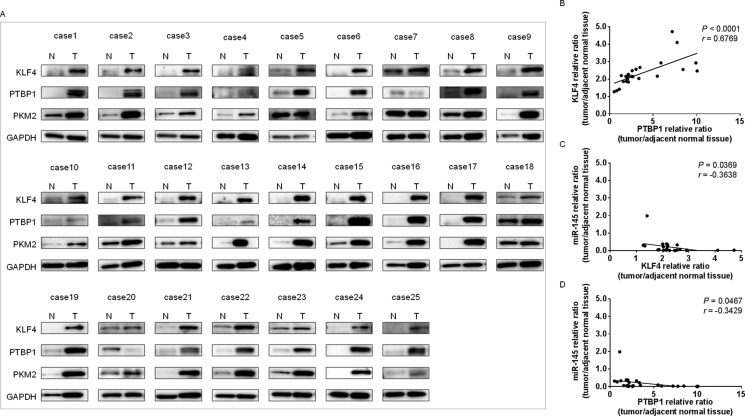
Increased expression levels of KLF4, PTBP1, and PKM2 in tumor samples from BC patients (**A**) KLF4, PTBP1, and PKM2 expression in 25 bladder cancer samples as determined by Western blot analysis. Details of the characteristics of the samples are given in Table 1. Densitometric analysis of KLF4 and PTBP1 were performed. GAPDH was used as the control. (**B**) Correlation between the relative expression levels of KLF4 and PTBP1. Levels are plotted as a scatter plot. (**C**) Correlation between the relative expression levels of miR-145 and KLF4. Levels are plotted as a scatter plot. (**D**) Correlation between the relative expression levels of miR-145 and PTBP1. Levels are plotted as a scatter plot.

Based on our results, we examined the expression levels of KLF4, PTBP1, and PKM2 in clinical BC samples by performing Western blot analysis on available protein samples. Surprisingly, all cases showed an increased KLF4 level by using both antibodies of CST (case1~15) and Proteintech (case16~25) (Figure 7A). Moreover, most of the samples tested showed increased PTBP1 and PKM2 as well. Also, KLF4 expression was highly correlated with that of PTBP1 (*r* = 0.6769, *P* < 0.0001, Figure 7B); KLF4 expression was inversely correlated with that of miR-145 (*r* = −0.3638, *P* = 0.0369, Figure 7B); as was PTBP1 expression (*r* = −0.3429, *P* = 0.0467, Figure 7C). These findings suggested that KLF4 plays an important role in promoting cell growth, at least in part through the maintenance of the Warburg effect in BC cells.

**Figure 8:**
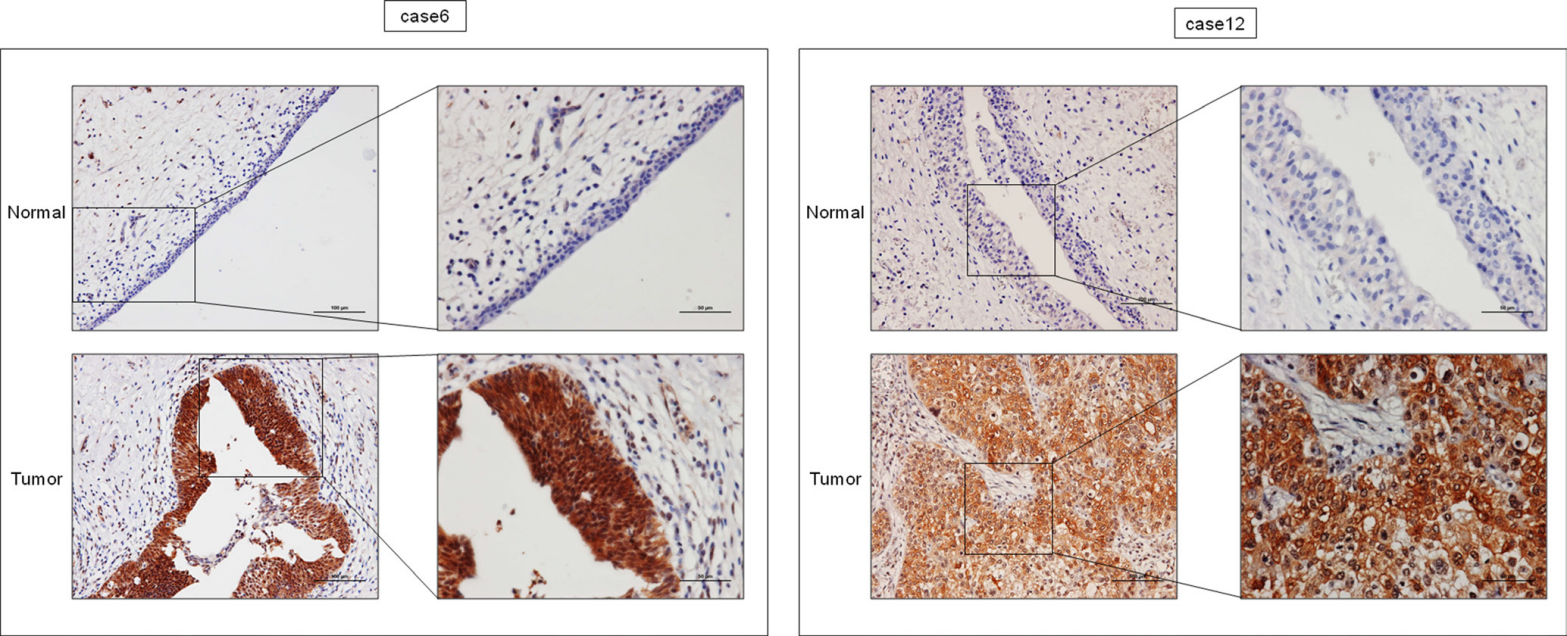
Expression levels of KLF4 were up-regulated in tumor tissues of BC samples (cases 6 and 12) as determined by immunohistochemical staining Representative images of tumor and adjacent unaffected tissue are shown. Tumor tissues stained positive, while no staining remained negative in the adjacent unaffacted tissue. A bar scale is located in the picture.

Furthermore, we examined immunohistochemical stainings to evaluate KLF4 expression in KLF4 positive clinical samples by Western blot analysis in bladder cancer (5 samples). As shown in Figure 8, all samples showed KLF4-positive staining in the tumor and KLF4-negative staining in adjacent normal tissue. We concluded that intratumor KLF4 expression is increased in these cases, as evaluated by both Western blot analysis and immunohistochemical staining.

### KLF4 contributed to both c-Myc and PTBP1 expression

**Figure 9:**
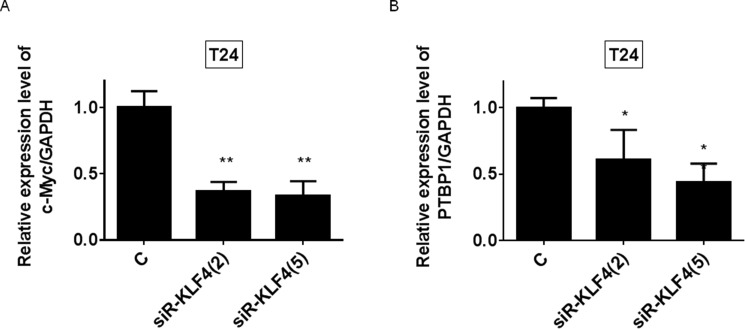
KLF4 positively regulated the c-Myc and PTBP1 expression (**A** and **B**) mRNA expression levels of c-Myc and PTBP1 at 48h after transfection of T24 and 253J B-V cells with siR-KLF4 (2, 5 nM).

We furthermore examined the effect of silencing KLF4 on mRNA levels of c-Myc and PTBP1 to define the relationship between KLF4 and c-Myc or PTBP1. As shown in Figure 9A and 9B, silencing KLF4 decreased the mRNA expression levels of c-Myc and PTBP1, respectively. The results suggested that KLF4 plays a pivotal role in the maintenance of the Warburg effect via c-Myc and/or PTBP1 and is a crucial gene in miR145/Warburg effect pathways.

## DISCUSSION

In the current study, we show that miR-145 was significantly decreased in BC clinical samples compared with its expression in NBEs (Figure 1A) and that the ectopic expression of miR-145 inhibited cell growth in the BC cell lines tested (Figure 2A). We thus confirmed that miR-145 acts as an anti-oncomiR in BC cells.

We demonstrated that miR-145 regulates the Warburg effect through the miR-145/KLF4/PTBP1/PKMs axis. PTBP1 promotes expression levels of cancer-dominant PKM2 by including exon 10 of the PKM gene to produce this isoform [12]. So far, several transcriptional factors such as c-Myc, STAT 3, and E2F1 have been proposed as PTBP1 regulators [22, 25]. Recently, we reported that miR-124-mediated switching of PKM isoform expression from cancer-dominant PKM2 to PKM1 disrupted the Warburg effect in colon cancer cells [23]. Also, we showed that Imatinib and AIC-47 disrupt the Warburg effect through modulation of *BCR-ABL* expression in leukemic cells [29]. Hence, we believe that the PKM2/PKM1 ratio is one of the essential factors affecting cancer cell growth.

**Figure 10:**
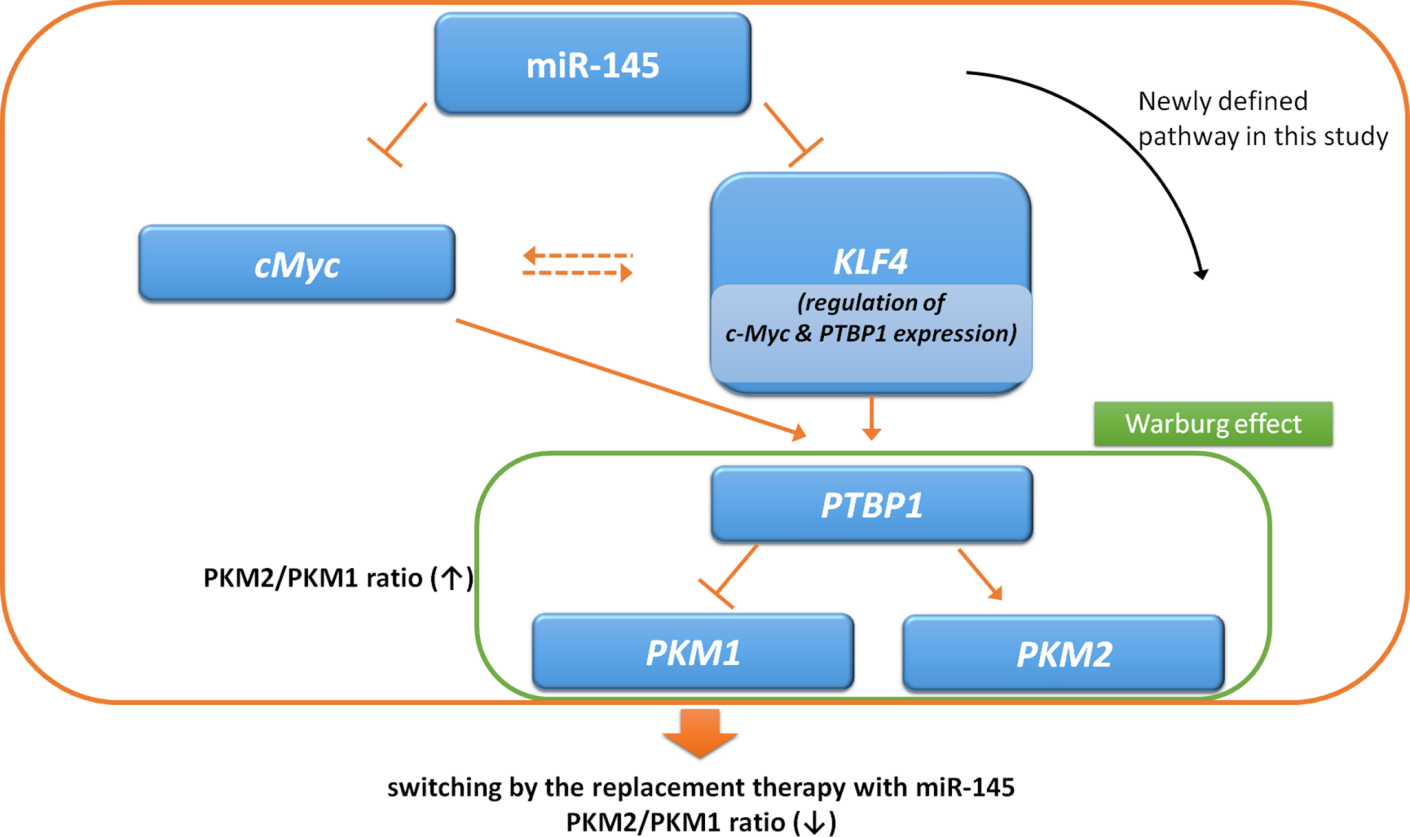
Schematic diagram of dual pathways, miR-145/KLF4/PTBP1/PKMs axis and miR-145/c-Myc/PTBP1/PKMs axis MiR-145 downregulates KLF4 and c-Myc in bladder cancer, and KLF4 and c-Myc directly regulate the expression of PTBP1. Silencing KLF4 suppresses mRNA levels of c-Myc and PTBP1.

We showed that either exogenous miR-145 or gene silencing of KLF4 decreased PTBP1, resulting in a switching of PKM isoforms from PKM2 to PKM1 (Figures 2B and 5B). As shown in Figure 3F, gene silencing of PTBP1 remarkably decreased the PKM2/PKM1 ratio, moreso than gene silencing of KLF4 or c-Myc. These results indicate that KLF4 and c-Myc act upstream of PTBP1/PKMs. Of note, judging from the results on the PKM2/PKM1 ratio (Figures 3C and 5C), regulation through the c-Myc/PTBP1 axis seemed to be more important than through the KLF4/PTBP1 axis. Moreover, gene silencing of c-Myc or KLF4 reduced the expression level of c-Myc or KLF4, respectively (Figures 3B and 5B), and silencing KLF4 decreased the mRNA expression of c-Myc and PTBP1 (Figure 9A and 9B). These results indicate that KLF4 plays an important role in the Warburg effect, as KLF4 affects the mRNA expression of both c-Myc and PTBP1 (Figure 10). We considered that KLF4 might be a transcription factor of c-Myc, and performed a ChIP assay. However, we could not obtain positive data (data not shown). Further investigation will be needed to clarify the exact relationship between c-Myc and KLF4 with regards to the Warburg effect. Interestingly, a different group recently reported that miR-145 targets hexokinase 2, which regulates the first step of glycolysis, in renal cancer cells [30]. These findings furthermore suggest that miR-145 deeply contributes to impairing the Warburg effect.

KLF4 plays a pivotal role in maintaining the self-renewal of murine embryonic stem cells [31]. Moreover, KLF4 also functions to reprogram mouse fibroblasts to become pluripotent stem cells, implying a role for KLF4 in maintaining the characteristics of stem cells [28]. Many groups reported that KLF4 acts as an anti-oncogene against various cancers including bladder [32, 33], colorectal [34, 35], gastric [36], pancreas [37], esophageal [38], lung [39], prostate [40], and hepatocellular carcinoma [41]. On the other hand, it was reported that KLF4 acts as an oncogene in primary breast cancer and oral and dermal squamous cell carcinoma cells [42–45]. The expression levels of KLF4 were increased in the clinical cases tested. As shown in Figure 7B and 7C, the expression levels of KLF4 were inversely correlated with the expression level of miR-145 and were strongly correlated with the expression levels of PTBP1. In addition, the expression levels of KLF4, as assessed by immunohistochemical staining, were increased in tumor regions compared to normal tissue, as shown in Western blot analysis (Figure 8). These findings suggest that KLF4 is likely to positively contribute to carcinogenesis in certain types of BC. Moreover, siR-KLF4 induced growth inhibition through induction of apoptosis in BC cells. Furthermore, based on searching databases such as Target Scan, KLF4 is targeted by representative anti-oncomiRs such as miR-7 [46], miR-34, and miR-124. Of note, miR-124 has 3 binding sites in the 3′-UTR of KLF4. MiR-124 is one of the negative regulators of the Warburg effect, as evidenced by our previous study on silencing PTBP1 [23].

Next, we examined intracellular levels of lactate, the end product of the glycolysis pathway, and ATP. Since with an impaired Warburg effect energy metabolism changes from glycolysis to oxidative phosphorylation with the TCA cycle, we expected ATP levels to be elevated. However, the ectopic expression of miR-145 increased lactate production significantly (Figure 6A). Metabolome analysis by Soga *et al*. [47] previously revealed the further activation of glycolysis as well as oxidative phosphorylation. Even if decrease of PTBP1 induced the switching from PKM2 to PKM1, the remaining PKM2 would fully work to further promote glycolysis. We hypothesize that miR-145 regulates the Warburg effect through silencing both KLF4 and c-Myc. Therefore, exogenous miR-145 would affect the Warburg effect more than gene silencing of KLF4. Moreover, the ectopic expression of miR-145 increased the ATP level significantly by causing the switching from PKM2 to PKM1 (Figure 6B). Since apoptosis requires ATP [48], the increase in the intracellular ATP levels after the ectopic expression of miR-145 may have contributed to the induction of apoptosis.

Finally, over the past few decades the standard therapy for non-muscle invasive bladder cancer (NMIBC) has been intravesical instillation of BCG (Bacille de Calmette et Guérin). We have already reported that intravesical administration of exogenous miR-145 exhibits an anti-tumor effect in a human bladder cancer xenograft model [11]. We are thus now aiming to compare the efficacy of intravesical delivery of exogenous miR-145 to that of BCG in the xenografted model mice.

## MATERIALS AND METHODS

### Patients and samples

All human samples were obtained from patients who had undergone procedures for biopsy or bladder cancer resection at Osaka Medical College Hospital (Takatsuki, Osaka, Japan). Informed consent in writing was obtained from each patient. Collection and distribution of the samples were approved by each of the appropriate institutional review boards in accordance with the Declaration of Helsinki. 28 patients with previously untreated (or recently diagnosed) bladder cancer were selected. The distribution according to other clinical parameters is shown in Table 1. Under a pathologist's supervision, all tissue sample pairs were collected from surgically resected tissues, with paired samples from the primary tumor and its adjacent non-tumor tissue in the same patient.

**Table 1:** Clinicopathological features of patients with bladder cancer

Case	Age	Sex^a^	Size^b^	Grade	Tstage	miR-145^c^
1	63	M	5	G2	Ta	D
2	85	M	5	G3	T1	ND
3	51	M	15	G3	T1	D
4	68	M	1	G1	Ta	D
5	79	F	2	G2	T1	D
6	33	M	4	G2	T1	D
7	72	M	4	G3	T1	D
8	49	M	3	G3	T1	D
9	85	F	3	G1	Ta	D
10	82	M	2	G3	T1	D
11	74	F	4	G3	T2	D
12	71	M	3	G3	T1	D
13	67	M	5	G2	T1	D
14	66	M	1	G3	T1	ND
15	84	F	3	G2	T1	D
16	75	M	3	G1	Ta	D
17	60	M	4	G1	Ta	D
18	86	M	20	G3	T1	D
19	62	M	3	G3	T1	D
20	75	M	4	G2	T2	D
21	58	F	2	G3	T1	D
22	76	M	5	G1	T1	D
23	82	M	4	G2	T1	D
24	77	M	1	G1	T1	D
25	71	F	3	G3	Ta	D
26	65	F	2	G2	T2	D
27	63	M	3	G3	T1	D
28	72	M	4	G3	T1	D

^a^M,male; F,female.

^b^Diameter in cm.

^c^D,down-regulation of miR-145 relative ratio (tumor tissue/adjacent normal tissue < 0.67).

ND, no down-regulation of miR-145 relative ratio (tumor tissue/adjacent normal tissue ≧ 0.67).

### Cell culture and cell viability

All cell lines were obtained from the JCRB (Japanese Collection of Research Bioresources) Cell Bank. All cell lines were cultured in RPMI-1640 medium supplemented with 10% (v/v) heat-inactivated FBS (Sigma-Aldrich Co, St. Louis, MO, USA) and 2 mM L-glutamine under an atmosphere of 95% air and 5% CO2 at 37°C. The number of viable cells was determined by performing the trypan-blue dye exclusion test.

### Transfection experiments

T24 cells or 253J B-V cells were seeded in 6-well plates at a concentration of 0.5 × 10^5^ per well (10–30% confluence) on the day before the transfection. The mature type of miR-145 (mirVana^TM^ miRNA mimic; Ambion, Foster City, CA, USA) was used for the transfection of the cells, which was achieved by using cationic liposomes, Lipofectamine^TM^ RNAiMAX (Invitrogen), according to the manufacturer's protocol. The nonspecific control miRNA (HSS, Hokkaido, Japan) sequence was 5′-GUAGG AGUAGUGAAAGGCC-3′, which was used as a control for nonspecific effects [5]. The sequence of the mature type of miR-145 used in this study was 5′-GUCCAGUUU UCCCAGGAAUCCCUU-3′; that of siR-KLF4, 5′-UUC AAGGGAAUUCUGGUCUUCCCUC-3′; that of siR-PTBP1, 5′-AUCUCUGGUCUGCUAAGGUCACUUC-3′; and that of siR-c-Myc, 5′-UUUGUGUUUCAACUGU UCUCGUCGU-3′. The effects manifested by the introduction of miR-145 into the cells were assessed 48 h after the transfection.

### Western blotting

Protein extraction and Western blotting experiments were performed as described in our previous publications [9, 49]. The following primary antibodies were used: antibodies against KLF4 (Cell Signaling Technology, Inc., Danvers, MA, USA and Proteintech Group, Inc, Rosemont, IL, USA), c-Myc, PTBP1, PARP, LC3B, p-PKM2, GAPDH (Cell Signaling Technology, Inc., Danvers, MA, USA), PKM1, and PKM2 (Novus Biologicals, USA). HRP-conjugated goat anti-rabbit and horse anti-mouse IgG (Cell Signaling Technology) were used as secondary antibodies. GAPDH served as an internal control.

### Real-time reverse transcription-PCR

Total RNA was isolated from cultured cells or tumor tissues by using a NucleoSpin miRNA isolation kit (TaKaRa, Otsu, Japan). RNA concentrations and purity were assessed by UV spectrophotometry. RNA integrity was checked by formaldehyde gel electrophoresis. To determine the expression levels of miR-145, we conducted quantitative RT-PCR (qRT-PCR) by using TaqMan MicroRNA Assays (Applied Biosystems) and THUNDERBIRD Probe qPCR Mix (TOYOBO Co., LTD., Osaka, Japan) according to the manufacturer's protocols. *RNU6B* was used as an internal control. For determination of the expression levels of DDX6 and glyceraldehyde-3-phosphate dehydrogenase (GAPDH) mRNAs, total RNA was reverse-transcribed with a PrimeScript® RT reagent Kit (TaKaRa). Real-time PCR was then performed with specific primers by using THUNDERBIRD SYBR qPCR Mix (TOYOBO). The primers for c-Myc, PTBP1, and GAPDH were the following: c-Myc-sense, 5′-TTC GGG TAG TGG AAA ACC AG-3′; c-Myc-antisense, 5′-CAG CAG CTC GAA TTT CTT CC-3′; PTB1-sense, 5′-ATC AGG CCT TCA TCG AGA TGC ACA-3′, and PTB1-antisense, 5′-TGT CTT GAG CTC CTT GTG GTT GGA-3′; GAPDH-sense, 5′-CCA CCC ATG GCA AAT TCC ATG GCA-3′, and GAPDH-antisense, 5′-TCT AGA CGG CAG GTC AGG TCC ACC-3′. GAPDH was used as an internal control. The relative expression levels were calculated using the ΔΔCt method.

### Luciferase reporter assay

By searching the Target Scan 6.2 database (http://www.targetscan.org/) to find algorithm-based binding sites of miR-145, we found a predicted binding site to be at positions 278–284 in the 3′-UTR of KLF4 mRNA. The sequence region 2161–2520, containing the putative binding sequence of miR-145, was inserted into a pMIR-REPORT^TM^ Luciferase miRNA Expression Reporter Vector (Applied Biosystems) according the manufacturer's protocol. Moreover, we made another pMIR construct encompassing a mutated seed sequence for miR-145 (Wild type, GACTGGAA; mutant, GACGTCAA) by using a PrimeSTAR^®^ Mutagenesis Basal Kit (TaKaRa). The mutation of the vector was confirmed by sequence analysis. The pRL-TK *Renilla* Luciferase Reporter vector (Promega, Madison, WI, USA) was used as an internal control vector. 253J B-V cells were seeded into 96-well plates at a concentration of 0.1 × 10^4^ per well on the day before the transfection. The cells were co-transfected with either reporter vector (0.01 μg/well each) and 20 nM miR-145 or nonspecific non-coding siRNA (Dharmacon, Tokyo, Japan). Luciferase activities were measured at 24 h after co-transfection by using a Dual-Glo Luciferase Assay System (Promega) according to the manufacturer's protocol. Luciferase activities were reported as the firefly luciferase/Renilla luciferase ratio.

### Lactate assay

Cells were incubated with miR-145 or miR-145 + antagomiR-145 for 48 h. Intracellular L-lactate was extracted by using an L-Lactate Assay kit (Cayman Chemical Company, Ann Arbor, MI, USA). L-lactate production was measured with a Lactate Colorimetric/Fluorometric Assay kit (Biovision, Milpitas, CA, USA) according to the manufacturer's instructions. Lactate production was normalized to cell numbers.

### ATP assay

To measure the ATP levels before commitment to programmed cell death, we incubated cells with miR-145 or miR-145 + antagomiR-145 for 48 h. ATP production was measured with an ATP Determination Kit (A22066; Invitrogen) according to the manufacturer's instructions. ATP production was normalized to cell numbers.

### Hoechst33342 staining

T24 cells or 253J B-V cells were collected at 48 h after transfection with miR-145 (20 nM). The details of the experimental protocol were given in a previous report [10, 50]. The number of apoptotic cells among 500 cells was counted.

### Immunostaining

253J B-V cells were incubated with miR-145 for 48 h and then immunostained with PKM2 antibody according to the immunofluorescence protocol of Cell Signaling Technology. The nuclei were stained with Hoechst 33342, and for actin labeling the cells were incubated with the fluorescent F-actin probe Rhodamine Phalloidin (Cytoskeleton, Denver, CO, USA). The cells were viewed with a BIOREVO fluorescence microscope (Keyence, Osaka, Japan). Standard immunohistochemical staining in clinical samples was performed using anti-KLF4 antibody (Santa Cruz Biotechnology and Proteintech Group, Inc, Rosemont, IL, USA).

### 3D Spheroid colorimetric viability assay

The 3D spheroid colorimetric viability assay was performed according to the manufacturer's protocol provided in a Cultrex 3-D spheroid Colorimetric Viability Assay Reagent Kit (Trevigen, Inc., Gaithersburg MD). Approximately 3000 253J B-V cells were suspended in 1× Spheroid Formation ECM reagent and transfected with miR-145 (100 nM). Then a 50 μl aliquot of the cell suspension was added to each well of a 3D culture Qualified 96-Well Spheroid Formation Plate. After that, the plate was centrifuged at 200 × g for 3 minutes at room temperature in a swinging bucket rotor, after which it was incubated at 37°C in a tissue culture incubator for 72 h to promote spheroid formation. Next, 5 μl of MTT Reagent was added per well; and incubation was then continued at 37°C for 24 h. Then 50 μl of Detergent Reagent was added to each well, and incubation was continued for another 24 h at 37°C to solubilize cells and MTT formazan crystals that had formed. The absorbance at 570 nm was then read.

### Statistics

Experiments were performed in triplicates. In experiments on clinical samples, we calculated the relative miR-145 ratio of tumor/adjacent normal tissues. We defined the expression levels > 1.5 as upregulation and those < 0.67 as downregulation, which fold changes were obtained from the results of linear discriminant analysis of the miRNA expression patterns from many of our previous reports [7, 51]. Statistical significances of differences were evaluated by performing the two-sided Student's *t-test*. The values were presented as the mean ± standard deviation. A *P value* < 0.05 was considered to be statistically significant.

## Abbreviations

BC: bladder cancer
mRNA: messenger RNA
miR: microRNA
PCR: polymerase chain reaction
KLF4: Kruppel-like factor 4
PKM: pyruvate kinase muscle
PTBP1: polypyrimidine tract-binding protein 1
siRNA: short interfering RNA
3′-UTR: 3′ untranslated region
